# Sensitivity of Human Mastadenovirus, the Causal Agent of Pharyngoconjunctival Fever, Epidemic Keratoconjunctivitis, and Hemorrhagic Cystitis in Immunocompromised Individuals, to Brincidofovir

**DOI:** 10.1128/spectrum.01569-21

**Published:** 2022-02-16

**Authors:** Nozomu Hanaoka, Masatoshi Hazama, Koji Fukushima, Tsuguto Fujimoto

**Affiliations:** a Center for Emergency Preparedness and Response, National Institute of Infectious Diseases, Tokyo, Japan; b SymBio Pharmaceuticals Limited, Tokyo, Japan; Center for Research and Advanced Studies (CINVESTAV-IPN)

**Keywords:** brincidofovir, human mastadenovirus, half-maximal inhibitory concentration, novel type

## Abstract

Human mastadenovirus (HAdV), a linear double-stranded DNA (dsDNA) virus, is the causal agent of several diseases, including pharyngoconjunctival fever, epidemic keratoconjunctivitis, and hemorrhagic cystitis, in immunocompromised individuals. There are more than 100 reported types of adenoviruses, but the pathogenicity of many HAdVs remains unknown. Brincidofovir (BCV) is a hexadecyloxypropyl lipid conjugate of cidofovir (CDV) that is active against dsDNA viruses. Clinical effectiveness of BCV against certain HAdV species has been reported; however, its activity against novel HAdV types remains unknown. We investigated the half-maximal inhibitory concentration (IC_50_) values of BCV for novel HAdV types and found that the epidemic keratoconjunctivitis-associated HAdV-D54 prevalent in the Asian region was the most susceptible. The mean overall IC_50_ value of BCV was lower than that of CDV, indicating that BCV is effective against HAdVs, including the novel types.

**IMPORTANCE** We investigated the IC_50_ values of BCV for novel HAdV types and found that the epidemic keratoconjunctivitis-associated HAdV-D54 prevalent in the Asian region was the most susceptible. In addition, the mean overall IC_50_ value of BCV was lower than that of CDV, indicating that BCV is effective against HAdVs.

## OBSERVATION

Human mastadenovirus (HAdV), a linear double-stranded DNA (dsDNA) virus, is the causal agent of several diseases (Table 1) (1, 2). There are more than 100 reported types of adenoviruses (2), but the pathogenicity of many HAdVs remains unknown. Furthermore, regional variations in prevalent HAdV types have been reported (Table 1).

**TABLE 1:** Comparison of prevalent adenoviruses between Japan and the United States

Species	Type^*a*^	Subgroup	Type of infection^*b*^	Detection rate (%)	
				Japan^*c*^	USA^*d*^
A	12		Gastrointestinal, respiratory, urinary	0	0.3
	31			1	0.3
B	**3**	1	Keratoconjunctivitis, gastrointestinal, respiratory, urinary	24	22.3
	**7**			0	12.9
	21			0	1.6
	**11**	2	Gastrointestinal, respiratory, urinary	0.2	0.2
	14			ND^*e*^	4.1
	34			ND	0.2
	35			0	0.5
C	**1**		Respiratory, gastrointestinal, including hepatitis, urinary	15	14.7
	**2**			28.8	19.6
	**5**			6.7	3.4
	**6**			2.3	1.2
D	**8**		Keratoconjunctivitis, gastrointestinal	0.5	3.4
	9			0	ND
	15			ND	0.1
	19			0.6	0.2
	22			ND	0.1
	29			ND	0.1
	33			0	ND
	**37**			2.5	0.7
	46			0	ND
	**53**			0.5	0.6
	**54**			5.1	ND
	**56**			1.3	0.1
	**64**			0.6	ND
E	**4**		Keratoconjunctivitis, respiratory	5.2	13.4
F	41		Gastrointestinal	2.6	0.2
G	52		Gastrointestinal	ND	ND

a HAdV types highlighted in bold were used in this study.

b Infection types are from the report by Ghebremedhin (1).

c Approximately 7,200 cases in total. Data were obtained from the Infectious Agents Surveillance Report (April 2021) (https://www.researchgate.net/profile/Tsuguto-Fujimoto/publication/358421869_IASR_Adenovirus_2020/data/6201d9205bdf0f2ef854ba76/IASRAdenovirus-2020.pdf).

d Approximately 1,500 cases in total. Data were obtained from the CDC (https://www.cdc.gov/adenovirus/reporting-surveillance/natrs/surveillance-data.html).

e ND, not detected.

Brincidofovir (BCV) is a hexadecyloxypropyl lipid conjugate of cidofovir (CDV) that is active against dsDNA viruses (3). It shows rapid incorporation into plasma membranes, with efficient cellular uptake because of its lipid conjugates, resulting in reduced side effects both *in vivo* and *in vitro* (1). BCV was found to be clinically effective for the treatment of HAdV-related diseases in pediatric recipients of hematopoietic cell transplants and especially for the control of adenoviremia during the lymphopenic phase (4). Several *in vitro* analyses of BCV against certain HAdV species have been reported (5); however, its activity against novel HAdV types remains unknown.

We investigated the half-maximal inhibitory concentration (IC_50_) values of BCV for the globally prevalent HAdVs, listed in Table 1. The HAdVs were prepared using A549 cells, as described previously (6). The assay medium was prepared with BCV or CDV, with serial 2-fold dilutions at concentrations of 0.00001 to 5 μM or 0.001 to 500 μM, respectively. CDV was used as an antiadenoviral reagent and served as a positive control, whereas reagent-free wells were used as negative controls. Virus titers were determined via real-time quantitative PCR (qPCR), as described previously (7). Briefly, A549 cells were seeded in 96-well plates and grown to confluence. The cells were then infected with each HAdV at 1 × 10^5^ genome copies/well. Four wells were used for each concentration of reagent. Almost all of the HAdV types, except HAdV-8 and HAdV-54, showed complete cytopathic effects for A549 at day 7 postinfection. After 7 d of inoculation with reagents and viruses, the viruses were harvested, and the nucleic acids were extracted to calculate the growth rate, as described previously (6). Briefly, the plates were subjected to two freeze-thaw cycles, and the samples from four wells were collected into one tube. Samples were then centrifuged at 1,500 × *g* for 2 min. The cell pellet along with 200 μL of supernatant was collected, and DNA was extracted using the High Pure viral nucleic acid kit (Roche) according to the manufacturer’s instructions. To calculate the IC_50_ values for BCV and CDV for virus growth, virus copy numbers were determined via qPCR (7). Copy numbers were normalized by setting the negative-control values to 100%. Three independent experiments were performed, and the summarized IC_50_ results are shown in Table 2. The cytotoxic effects, including cell death and growth inhibition, of BCV and CDV on A549 cells were evaluated. Confluent monolayer and trypsin-treated floating cells were used to analyze the sensitivity of these reagents through microscopy or real-time qPCR using the TaqMan RNase P detection reagents kit (ABI).

**TABLE 2:** Summary of IC_50_ values for BCV and CDV

HAdV type	Species	IC_50_ (μM)^*a*^	
		BCV	CDV
1	C	0.003 ± 0.000	0.7 ± 0.0
2	C	0.003 ± 0.000	0.7 ± 0.0
3	B	0.003 ± 0.000	0.4 ± 0.0
4	E	0.007 ± 0.002	1.3 ± 0.3
5	C	0.006 ± 0.005	0.6 ± 0.1
6	C	0.004 ± 0.000	0.7 ± 0.0
7	B	0.005 ± 0.002	0.8 ± 0.1
8	D	0.002 ± 0.002	0.3 ± 0.1
11	B	0.004 ± 0.002	1.5 ± 0.0
37	D	0.005 ± 0.002	0.7 ± 0.0
53	D	0.003 ± 0.001	0.3 ± 0.0
54	D	0.001 ± 0.000	0.3 ± 0.1
56	D	0.003 ± 0.000	0.7 ± 0.0
64	D	0.004 ± 0.001	1.6 ± 0.1
79	B	0.004 ± 0.000	1.1 ± 0.1
81	D	0.003 ± 0.000	0.5 ± 0.0
85	D	0.003 ± 0.000	0.3 ± 0.1

a Values are average **±** standard deviation, calculated as the average of the data obtained from three independent experiments.

According to the IC_50_ values averaged from three independent experiments, epidemic keratoconjunctivitis-associated HAdV-D54, which is prevalent in the Asian region, was the most susceptible (IC_50_ = 0.001 μM). The concentrations of BCV and CDV that caused 50% growth inhibition in A549 host cells were 0.6 and 15.7 μM, respectively. The mean overall IC_50_ of BCV was 0.003 μM, approximately 200-fold lower than that of CDV (0.7 μM), indicating that BCV is a broadly effective agent against HAdVs.
